# Global stability analysis for a generalized delayed SIR model with vaccination and treatment

**DOI:** 10.1186/s13662-019-2447-z

**Published:** 2019-12-21

**Authors:** A. Elazzouzi, A. Lamrani Alaoui, M. Tilioua, A. Tridane

**Affiliations:** 1 LSI Laboratory, FP Taza, Department of MPI, Sidi Mohamed Ben Abdellah University, Taza, Morocco; 2 MAMCS Group, M2I Laboratory, FST Errachidia, Moulay Ismaïl University of Meknès, Errachidia, Morocco; 3 Department of Mathematical Sciences, United Arab Emirates University, Al Ain, United Arab Emirates

**Keywords:** 34D03, 92D30, SIR epidemic model, Distributed delay, Generalized nonlinear incidence, Vaccination, Treatment, Lyapunov function

## Abstract

In this work, we investigate the stability of an SIR epidemic model with a generalized nonlinear incidence rate and distributed delay. The model also includes vaccination term and general treatment function, which are the two principal control measurements to reduce the disease burden. Using the Lyapunov functions, we show that the disease-free equilibrium state is globally asymptotically stable if $\mathcal{R}_{0} \leq 1 $, where $\mathcal{R}_{0} $ is the basic reproduction number. On the other hand, the disease-endemic equilibrium is globally asymptotically stable when $\mathcal{R}_{0} > 1 $. For a specific type of treatment and incidence functions, our analysis shows the success of the vaccination strategy, as well as the treatment depends on the initial size of the susceptible population. Moreover, we discuss, numerically, the behavior of the basic reproduction number with respect to vaccination and treatment parameters.

## Introduction

Mathematical modeling has become a powerful and important tool to understand infectious disease dynamic behavior and to improve control of the disease in a population. These models are often described by many forms such as: *SI*, *SIS*, *SIR*, or *SIRS* models, where *S* stands for susceptible subpopulation, *I* is infected subpopulation, and *R* is recovered subpopulation. The progress of a disease in a population is dictated by the nature and the mode of transmission between infected and susceptible individuals. The mode of transmission is the method of transfer by which the infection moves or carries from one place to another to reach the new host (for example airborne, saliva, vector-borne, and bodily fluids). Hence, it is natural to adapt these models to the concerned disease by choosing the right incidence function. It is known that the function forms of the incidence rate of the infection have a crucial role in the modeling of the infection dynamics, many forms of incidence function have been considered by the researchers in mathematical epidemiology, for example, the bilinear incidence rate *βSI*, where *β* is the transmission rate of infection, the saturated incidence rate $\frac{ \beta \mathit{SI}}{1+ \alpha I}$, with *α* defined as the inhibitory coefficient, and many other forms (see [1–7]). To make a model more realistic, the introduction of the time delay is more interesting, and considerable attention has been paid by several authors to studying the dynamics of epidemic models with discrete or distributed time delay (see [3, 4, 8–11]).

Vaccination and treatment are the two main public health control strategies that help to minimize the burden of an infectious disease spread and to delay a possible outbreak. Vaccination has the role of preventing healthy people from getting infected by a disease, while treatment cures a disease and can also be used as a prophylactic. These control strategies are usually used together to contain the disease spread (see [12] in the context of influenza). Tulu et al., in [13], developed a mathematical model to study the effect of both vaccination and quarantine on the spread of Ebola virus, they applied the vaccination strategy to the susceptible individuals. However, in [14], the authors studied the global dynamics of an SEIRS epidemic model with preventive vaccination applied to the newborns. Various vaccination policies were studied in different mathematical models (see [8, 15–18]). It is well known in classical epidemic models that the recovery rate due to treatment is proportional to the number of the infected individuals. However, this proportionality is not satisfied in the reality because of limited medical facilities (see [19]). In order to include the limited capacity of medical resources, Chauhan et al., in [20], considered the piecewise linear treatment function of the form

1 $$ T(I)= \textstyle\begin{cases} kI &\mbox{if } 0\leq I \leq I_{0}, \\ kI_{0} &\mbox{if } I>I_{0}, \end{cases} $$

where $I_{0}$ is the capacity of treatment. Recently, Li introduced the following saturated treatment function [21]:

2 $$ T(I)=\frac{a I}{1+\epsilon I}, $$

where *a* represents the maximal medical resources supplied per united time and *ϵ* is half-saturation constant, which measures the effect of being delayed for treatment. Other works have investigated the effects of the treatment on an epidemic (see [19–25]) and also its optimal control (see [26]).

The motivation of this work comes from [10, 11], where the authors studied an SIR epidemic model with nonlinear incidence function, and from [19–21], where the authors considered a special type of treatment function. The present work would be a continuation and generalization of the above cited works. It is concerned with a generalized SIR epidemic model with distributed delay, vaccination, and treatment. This model incorporates distributed delay, general incidence function, vaccination, and general function treatment. In fact, we apply the vaccination to both susceptible and newborn individuals. On the newborn individuals, we apply the mechanism of “all-or-nothing” vaccine. Recall that an “all-or-nothing” vaccine offers complete protection to a subset of the vaccinated individuals, but the remainder of them stays susceptible to catching the disease. Second, we consider a class of treatment functions satisfying suitable conditions, and it is more general than the one given by (1) or (2). Moreover, it is necessary to point out that the delay in this model represents the incubation time taken to become infectious. This model can be applied to investigate the impact of the vaccination and the treatment in containing the spread of infections which have an incubation time to become infectious, for example, SARS-CoV(see [27, 28]). Our purpose in this work is to investigate the impact of the combined vaccination and treatment strategies on the dynamic behavior of the considered model. We prove that the basic reproduction number $\mathcal{R}_{0}$ depends explicitly on the vaccination parameters and the general treatment function $T(I)$. Moreover, we discuss the global stability of the model near equilibria (the disease-free equilibrium $E_{0}$ and the disease-endemic equilibrium $E^{*}$) by means of $\mathcal{R}_{0}$ and Lyapunov’s method. Furthermore, to verify the theoretical results, numerical simulations are performed for special treatment and incidence functions. For illustration, we give some numerical results on the behavior of the basic reproduction number $R_{0}$ with respect to vaccination and treatment parameters.

The paper is organized as follows. We give a mathematical model formulation in Sect. 2. In Sect. 3, we propose a mathematical analysis of the considered model. More precisely, we calculate the basic reproduction number $\mathcal{R} _{0}$, and we determine the disease-free equilibrium $E_{0}$ and the endemic equilibrium $E^{*}$. Moreover, we prove the local stability of the disease-free equilibrium and the global stability of $E_{0}$ and $E^{*}$. In Sect. 4, we give some numerical examples with an incidence and treatment functions satisfying assumptions presented in the previous sections. We finish the paper, in the last section, by providing some concluding remarks.

## Mathematical model and preliminaries

**Figure 1:**
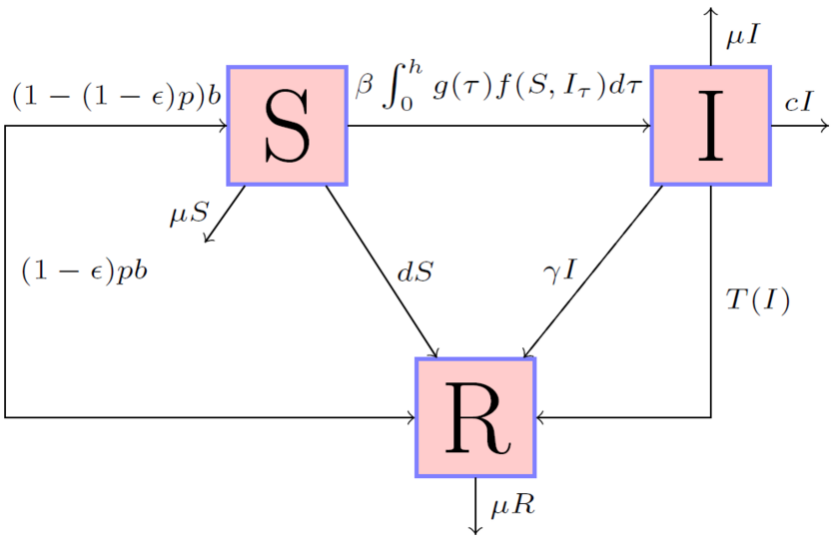
Flow diagram of the disease transmission

In this work, we are interested in a general SIR epidemic model with distributed delay, vaccination, and treatment. The dynamics are governed by the diagram in Fig. 1.

**Figure 2:**
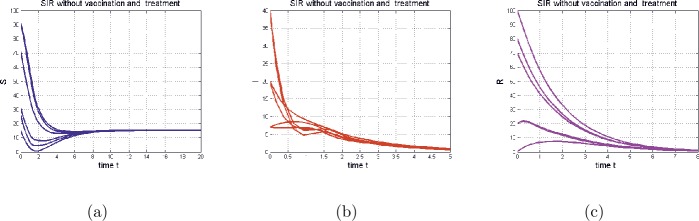
The time series of model (3) in the special case (12), with Figures (**a**), (**b**), and (**c**) representing (respectively) $S(t)$, $I(t)$, and $R(t)$. The parameters of the model are $b = 10$, $\mu = 0.65 $, $\beta = 0.2 $, $c = 0.77 $, $\gamma = 0.75 $, $h=1.5$, $d=0$, $p=0$, $\epsilon =0$, $\xi = 10$, and $a=0$. In this case $\overline{\mathcal{R}_{0}}=1.4179> 1$

From Fig. 1, we have the following SIR model:

3 $$ \textstyle\begin{cases} \frac{dS(t)}{dt}= (1-(1-\epsilon )p)b- (\mu +d) S(t)- \beta \int _{0}^{h}g(\tau )f(S(t),I(t-\tau ))\,d\tau , \\ \frac{dI(t)}{dt}= \beta \int _{0}^{h}g(\tau )f(S(t),I(t-\tau ))\,d\tau -(\mu +c+\gamma )I(t)-T(I), \\ \frac{dR(t)}{dt}=T(I)+(1-\epsilon )pb+\gamma I(t)+d S(t)- \mu R(t), \end{cases} $$

where $S(t) $, $I(t)$, and $R(t) $ denote the numbers of susceptible, infective, and recovered individuals at time *t* respectively. The susceptibles are augmented by the birth of newborns. Here, we assume that the birth rate *b* and death rate *μ* are not the same. The parameter *p* is the fraction of the vaccinated newborns. A fraction $\epsilon \in [0.1)$ (the all-or-nothing parameter) of the vaccinated newborns exhibits an unsuccessful vaccination and passes directly to the susceptible class. Our vaccine has an efficacy of $1 - \epsilon $ (see [27–33]). For simplicity, we assume that the recovered class stands also for the vaccinated state. Hence, susceptible individuals get vaccinated with rate *d*.

The nonlinear incidence rate and distributed delay are considered to represent wide class epidemic model similarly as in [10, 11]. More precisely, by taking *β* the disease transmission coefficient, individuals leave the susceptible class at a rate $\int _{0}^{h}g(\tau )f(s(t),i(t-\tau ))\,d\tau $, where *h* represents the maximum time taken to become infectious. The function *g* that satisfies $\int _{0}^{h}g(\tau )\,d\tau = 1$ is assumed to be nonnegative.

The function $f:\mathbb{R}^{2}_{+} \rightarrow \mathbb{R}^{2}_{+} $ is assumed to be continuously differentiable in the interior of $\mathbb{R}^{2}_{+} $ such that

$$ f(0, I) = f(S, 0) = 0\quad \mbox{for } S, I\geqslant 0, $$

and the following hypotheses hold:

$( \mathbf{H}_{1}) $
$f(S,I) $ is a strictly monotone increasing function of $S \geqslant 0 $ for any fixed $I > 0$ and a monotone increasing function of $I > 0 $ for any fixed $S \geqslant 0$.
$(\mathbf{H}_{2}) $
$\phi (S, I) = \frac{f(S,I )}{I} $ is a bounded and monotone decreasing function of $I > 0 $ for any fixed $S \geqslant 0 $ and $k(S) = \lim_{I \rightarrow 0^{+}} \phi (S, I) $ is a continuous and monotone increasing function on $S \geqslant 0 $.

We also assume that the disease causes death with rate *c* and *γ* is the natural recovery rate of the infected individuals.

The function $T: \mathbb{R}_{+} \rightarrow \mathbb{R}_{+}$ represents the treatment function which we assume to be continuously differentiable and concave down satisfying the following hypotheses:

$(\mathbf{T}_{1})$
$T(0) = 0$.
$(\mathbf{T}_{2})$
The treatment rate $\frac{T(I)}{I}$ is monotone increasing.

The assumption of the concavity of the treatment function refers to the fact that the supply of the treatment drugs increases as the disease kicks off in the population until it reaches a maximum level, then the treatment drug stocks start going down due to the exhaustive consumption.

Hypothesis $(\mathbf{T}_{1})$ means that there is no treatment if there is no infection, while hypothesis $( \mathbf{T}_{2})$ reflects the increasing effort needed from the public health authorities to provide treatment during the time of the infections.

The initial condition for the above system is given for $\theta \in [-h,0] $ by

4 $$ S( \theta ) = \Phi _{1} ( \theta ),\qquad I( \theta ) = \Phi _{2} ( \theta ) \quad \mbox{and}\quad R( \theta ) = \Phi _{3} ( \theta ), $$

with $\Phi = (\Phi _{1}, \Phi _{2}, \Phi _{3}) \in C^{+}$. The space of continuous functions from $[-h,0]$ to $\mathbb{R}^{2}$ provided with the uniform topology is $C:= C([-h,0],\mathbb{R}^{3})$, and $C^{+} = C([-h,0],( \mathbb{R}^{3})^{+}) $ is the nonnegative cone of *C*. Let $\Phi _{i}( \theta ) \geq 0$, $i=1,2,3$, for $\theta \in [-h,0] $.

Following the standard approach (see [34, 35]), model (3) has a unique local solution, i.e., for all $t \in [ 0, \delta ] $, $\delta \geq 0$. Moreover, we have the following preliminary results.

### Proposition 2.1

*The solution of* (3), *with initial condition* (4), *is positive and bounded*.

### Proof

We prove, by contradiction, that the solution $(S(t),I(t),R(t)) $ is positive. Let $t_{1} = \min \{t\geq 0: S(t)I(t) =0 \} $, and we assume that $S(t_{1}) =0 $, which implies that, for all $0 \leq t \leq t_{1} $, $I(t) \geq 0 $. Let

$$ \zeta = \min_{ 0 \leq t \leq t_{1}} \biggl\{ \frac{(1-(1-\epsilon )p)b}{S(t)} - (\mu +d) - \beta \int _{0}^{h} g(\tau ) \frac{f(S(t),I(t-\tau ))}{S(t)}\,d \tau \biggr\} . $$

It follows that

$$ \frac{dS(t)}{dt} \geq \zeta S(t), $$

then

$$ S(t_{1}) \geq S(0) \exp (\zeta t_{1}) > 0 . $$

This contradicts $S(t_{1}) = 0 $. Using a similar argument, we can prove that $S(t) > 0 $ and $I(t) > 0 $ for all $t \geq 0 $. The positivity of *R* follows from the inequality

$$ \frac{dR(t)}{dt} \geq - \mu R(t), $$

which implies that

$$ R(t) \geq R(0) \exp (- \mu t) > 0 . $$

For the boundedness, we note that

$$ \frac{dn(t)}{dt}= \mu \biggl(\frac{b}{\mu }-n(t) \biggr). $$

It follows that $\lim_{t \rightarrow + \infty } n(t)= \frac{b}{ \mu } $, which completes the proof. □

The local existence and boundedness of the solution of (3) imply the global existence of the solution.

As the variable *R* does not appear in the first two equations for system (3), we focus our analysis on the reduced system

5 $$ \textstyle\begin{cases} \frac{dS(t)}{dt}= (1-(1-\epsilon )p)b- (\mu +d) S(t)- \beta \int _{0}^{h}g(\tau )f(S(t),I(t-\tau ))\,d\tau , \\ \frac{dI(t)}{dt}= \beta \int _{0}^{h}g(\tau )f(S(t),I(t-\tau ))\,d\tau -(\mu +c+\gamma )I(t)-T(I). \end{cases} $$

## Analysis of the model

### Existence of equilibria points

System (5) has a disease-free equilibrium

6 $$ E_{0} = (S_{0}, 0),\quad \mbox{with } S_{0} = \frac{(1-(1-\epsilon )p)b}{\mu +d}. $$

On the other hand, using the next generation method [36], the basic reproduction number should be as follows.

#### Lemma 3.1

*The basic reproduction number is*

$$ {\mathcal{R}}_{0}=\frac{\beta k (\frac{(1-(1-\epsilon )p)b}{( \mu +d)} )}{(\mu +\gamma +c)+T^{\prime }(0)} = \frac{ \beta k(S_{0})}{( \mu +\gamma +c)+T^{\prime }(0)}. $$

*Note that* $S_{0} $*depends on the vaccination of susceptible population and the treatment terms*.

#### Proof

Let $X=(I,S)^{T}$, then it follows from system (5) that

$$\begin{array}{rl} \frac{dX}{dt}= & \left(\begin{array}{c} \beta \int_{0}^{h}g(\tau )f(S(t),I(t-\tau ))\;d\tau \\ 0 \end{array}\right)\\ & -\left(\begin{array}{c} \left(\mu +c+\gamma )I(t)+T(I\right)\\ \beta \int_{0}^{h}g(\tau )f(S(t),I(t-\tau ))\;d\tau -(1-(1-\epsilon )p)b+(\mu +d)S(t) \end{array}\right),\\ = & F-\nu . \end{array}$$

The Jacobian of matrices $\mathcal{F}$ and *ν* at the disease-free equilibrium $E_{0}$ is given by

$$F=\left(\begin{array}{cc} \beta f_{2}(E_{0}) & 0\\ 0 & 0 \end{array}\right)\;\text{and}\;V=\left(\begin{array}{cc} \left(\mu +c+\gamma )+T^{\prime }(0\right) & 0\\ \beta f_{2}(E_{0}) & \mu +d \end{array}\right),$$

where $f_{2}(E_{0})$ is the derivative of *f* with respect to *I* at $E_{0}$. The inverse of *V* is given by

$$V^{-1}=\left(\begin{array}{cc} \frac{1}{\left(\mu +c+\gamma )+T^{\prime }(0\right)} & 0\\ \frac{-\beta f_{2}(E_{0})}{\left((\mu +c+\gamma )+T^{\prime }(0))(\mu +d\right)} & \frac{1}{\mu +d} \end{array}\right).$$

Thus, the next generation matrix for system (5) is

$$FV^{-1}=\left(\begin{array}{cc} \frac{\beta f_{2}(E_{0})}{\left(\mu +c+\gamma )+T^{\prime }(0\right)} & 0\\ 0 & 0 \end{array}\right).$$

Since $R_{0}$ is the spectral radius of the matrix $FV^{-1}$, it follows that the basic reproduction number is

$$ \mathcal{R}_{0}=\frac{\beta f_{2}(E_{0})}{(\mu +\gamma +c)+T^{\prime }(0)} = \frac{ \beta k(S_{0})}{(\mu +\gamma +c)+T^{\prime }(0)}. $$

 □

To prove the existence of an endemic equilibrium, we need the following lemma.

#### Lemma 3.2

*Assume that assumptions* $(\mathbf{T}_{1})$*and* $( \mathbf{T}_{2})$*are satisfied*. *Then the equation*

$$ b-a u-T(u)=0, $$

*for* $a>0$*and* $b>0$, *has a unique positive solution*.

#### Proof

Let $\mathcal{K}$ be the function defined on $\mathbb{R_{+}}$ by

$$ \mathcal{K}(u)=b-a u-T(u). $$

We have

$$ \mathcal{K}(0)=b>0 \quad \mbox{and} \quad \mathcal{K}\biggl(\frac{b}{a} \biggr)=-T \biggl( \frac{b}{a}\biggr)< 0. $$

Since $\mathcal{K}$ is continuous, the equation $\mathcal{K}(u)=0$ has a unique positive solution in the interval $(0,\frac{b}{a})$. □

Next result shows the existence of the endemic equilibrium.

#### Theorem 3.1

*Assume that assumptions* $(\mathbf{H}_{1})$, $(\mathbf{H}_{2})$, $(\mathbf{T}_{1})$, *and* $(\mathbf{T}_{2})$*hold*. *If* $\mathcal{R}_{0} > 1 $, *then system* (5) *admits a unique endemic equilibrium* $E^{*} = (S^{*}, I^{*}) $.

#### Proof

At the equilibrium point, we have

$$ \bigl(1-(1-\epsilon )p\bigr)b- (\mu +d) S^{*} -(\mu +c+\gamma )I^{*} -T\bigl(I^{*}\bigr)=0, $$

and so

$$ S^{*}=\frac{(1-(1-\epsilon )p)b-(\mu +c+\gamma )I^{*}-T(I^{*})}{ \mu +d}. $$

Let $\overline{\mathcal{K}} $ be the function defined for $\mathbb{R}^{+}\setminus \{0\} $ to $\mathbb{R}$ by

$$ \overline{\mathcal{K}}(I)=\beta \frac{f (S^{*},I )}{I}-(\mu +c+ \gamma )- \frac{T(I)}{I}. $$

By hypotheses $(\mathbf{H}_{2})$ and $(\mathbf{T}_{2})$, $\overline{ \mathcal{K}}$ is strictly monotone decreasing on $\mathbb{R}^{+} \setminus \{0\}$ satisfying

$$ \lim_{I \rightarrow 0^{+}} \overline{\mathcal{K}}(I)=\beta k \biggl( \frac{(1-(1-\epsilon )p)b}{\mu +d} \biggr)-(\mu +c+\gamma )-T^{\prime }(0)= \bigl(\mu +c+ \gamma +T^{\prime }(0)\bigr) (\mathcal{R}_{0}-1)>0. $$

Moreover, by Lemma 3.2, there exists a unique solution $I^{0}$ of the following equation:

$$ \frac{(1-(1-\epsilon )p)b}{\mu +d}-\frac{1}{{\mu +d}}\bigl((\mu +c+ \gamma )I+T(I) \bigr)=0, $$

and then

$$ \overline{\mathcal{K}}\bigl(I^{0}\bigr)=-\biggl((\mu +c+\gamma )+ \frac{T(I^{0})}{I ^{0}}\biggr)< 0. $$

Hence, there exists a unique positive real $I^{*}$ such that

$$ 0 < I^{*} < I^{0}\quad \mbox{and} \quad \overline{\mathcal{K}} \bigl(I^{*}\bigr)=0, $$

which allows us to conclude that $E^{*} = (S^{*}, I ^{*})$ is the unique endemic equilibrium of system (5). □

### Local stability analysis

In this section, we discuss the local stability of the disease-free equilibrium of system (5). We have the following result.

#### Theorem 3.2

*If* $\mathcal{R}_{0} < 1 $, *then the disease*-*free equilibrium* $E_{0} = (S_{0}, 0)$*is locally asymptotically stable*.

#### Proof

We consider the following linearization equation of system (5) at $E_{0}$:

7 $$ \textstyle\begin{cases} \frac{dS(t)}{dt}=- (\mu +d) S(t)- \beta \int _{0}^{h}g(\tau )f_{2}(E_{0})I(t-\tau )\,d\tau , \\ \frac{dI(t)}{dt}= \beta \int _{0}^{h}g(\tau )f_{2}(E_{0})I(t-\tau )\,d\tau -(\mu +c+\gamma )I(t)-T ^{\prime }(0)I(t). \end{cases} $$

Substituting $(S(t),I(t))=\exp (\lambda t) (S_{0},I_{0})$ into (7), we have

$$ \textstyle\begin{cases} \lambda S_{0} \exp (\lambda t) =- (\mu +d) S_{0} \exp (\lambda t)- \beta \int _{0}^{h}g(\tau )f_{2}(E_{0})I_{0} \exp \lambda ( t-\tau )\,d\tau , \\ \lambda I_{0} \exp (\lambda t)= \beta \int _{0}^{h}g(\tau )f_{2}(E_{0})I_{0} \exp \lambda ( t-\tau )\,d\tau -( \mu +c+\gamma +T^{\prime }(0))I_{0} \exp (\lambda t), \end{cases} $$

hence

8 $$ \textstyle\begin{cases} - (\mu +d +\lambda ) S_{0} - \beta \int _{0}^{h}g(\tau )f_{2}(E_{0})I_{0} \exp ( -\lambda \tau ) \,d\tau =0, \\ \beta \int _{0}^{h}g(\tau )f_{2}(E_{0})I_{0} \exp ( -\lambda \tau ) \,d\tau -( \mu +c+\gamma +T^{\prime }(0)+ \lambda )I_{0}=0. \end{cases} $$

We can write (8) in the following abstract form:

$$ BX=0, $$

where $X=(\begin{array}{c} S_{0}\\ I_{0} \end{array})$ and

$$ B= \begin{pmatrix} -(\mu +d+\lambda ) & - \beta f_{2}(E_{0}) \int _{0}^{h}g(\tau ) \exp ( -\lambda \tau ) \,d\tau \\ 0 & \beta f_{2}(E_{0}) \int _{0}^{h}g(\tau )\exp ( -\lambda \tau ) \,d\tau - (\mu +c+\gamma +T ^{\prime }(0) + \lambda ) \end{pmatrix} . $$

Then the characteristic equation of system (8) at $E_{0} $ is of the form

9 $$ (\mu +d +\lambda ) \biggl(- \beta f_{2}(E_{0}) \int _{0}^{h}g(\tau )\exp ( - \lambda \tau ) \,d \tau + \bigl(\lambda +\mu +c+\gamma +T^{\prime }(0)\bigr)\biggr)=0. $$

It is clear that $\lambda = -(\mu +d)$ is a root of (9). All other roots *λ* of (9) are determined by the following equation:

$$ - \beta f_{2}(E_{0}) \int _{0}^{h}g(\tau )\exp ( -\lambda \tau ) \,d \tau + \bigl(\lambda +\mu +c+T^{\prime }(0)+\gamma \bigr)=0. $$

Then by separating real $(\Re )$ and imaginary $(\Im )$ parts, we derive

$$ \textstyle\begin{cases} - \beta f_{2}(E_{0}) \int _{0}^{h}g(\tau ) \exp ( -\Re (\lambda ) \tau )\cos (\Im (\lambda ) \tau ) \,d\tau + (\Re (\lambda ) +\mu +c+\gamma +T^{\prime }(0))=0, \\ - \beta f_{2}(E_{0}) \int _{0}^{h}g(\tau )\exp ( -\Re (\lambda ) \tau )\sin (\Im (\lambda ) \tau ) \,d\tau + (\Im (\lambda ) +\mu +c+\gamma +T^{\prime }(0))=0. \end{cases} $$

Using the first equation of the above system, we obtain

10 $$ \Re (\lambda ) = \beta f_{2}(E_{0}) \int _{0}^{h}g(\tau ) \exp \bigl( - \Re (\lambda ) \tau \bigr)\cos \bigl(\Im (\lambda ) \tau \bigr) \,d\tau - \bigl(\mu +c+ \gamma +T^{\prime }(0)\bigr). $$

We suppose, by contradiction, that there exists $\lambda \in \mathbb{C}$ such that $\Re (\lambda )\geq 0$, and it satisfies equality (10). Then

11 $$ \beta f_{2}(E_{0}) \int _{0}^{h}g(\tau ) \exp \bigl( -\Re (\lambda ) \tau \bigr)\cos \bigl(\Im (\lambda ) \tau \bigr) \,d\tau \geq \mu +c+\gamma +T^{\prime }(0). $$

Since the function *T* is concave down, it follows that $T^{\prime }(0) \geq 0$.

Moreover, we know that $f_{2}(E_{0})>0$, which implies

$$ 0\leq \int _{0}^{h}g(\tau ) \exp \bigl( -\Re (\lambda ) \tau \bigr)\cos \bigl(\Im (\lambda ) \tau \bigr) \,d\tau \leq 1. $$

If $\mathcal{R}_{0} <1 $, then $\beta f_{2}(E_{0}) < \mu +c+\gamma +T ^{\prime }(0)$ and

$$ \beta f_{2}(E_{0}) \int _{0}^{h}g(\tau ) \exp \bigl( -\Re (\lambda ) \tau \bigr) \cos \bigl(\Im (\lambda ) \tau \bigr) \,d\tau < \mu +c+\gamma +T^{\prime }(0), $$

which gives a contradiction with inequality (11). Then the real parts of all the eigenvalues of (9) are negative. Therefore, if $\mathcal{R}_{0} < 1$, the disease-free equilibrium $E_{0} $ of system (5) is locally asymptotically stable. Now, let

$$ P(\lambda )= - \beta f_{2}(E_{0}) \int _{0}^{h}g(\tau )\exp ( -\lambda \tau ) \,d \tau + \bigl( \lambda +\mu +c+\gamma +T^{\prime }(0)\bigr). $$

From the fact that $P(0)= (\mu +c+\gamma +T^{\prime }(0))(1-\mathcal{R} _{0})<0 $ if $\mathcal{R}_{0} > 1 $ and $\lim_{\lambda \longrightarrow + \infty } P(\lambda )= + \infty $, we conclude that there is at least one positive root of (9). Hence, if $\mathcal{R}_{0}> 1$, $E_{0}$ is unstable. □

### Global stability of the disease-free equilibrium

The next result gives the condition of the global asymptotic stability of the disease-free equilibrium $E_{0}$ of system (5).

#### Theorem 3.3

*If hypotheses* $(\mathbf{H}_{1})$, $(\mathbf{H}_{2})$, $(\mathbf{T}_{1})$, *and* $(\mathbf{T}_{2})$*hold and* $\mathcal{R}_{0} \leq 1 $, *then the disease*-*free equilibrium* $E_{0} $*of system* (5) *is globally asymptotically stable*.

#### Proof

To prove this result, we consider the following Lyapunov function:

$$ V(t)=V_{1}(t)+I(t)+V_{2}(t)+V_{3}(t), $$

where

$$\begin{aligned}& V_{1}(t)= \int _{\frac{(1-(1-\epsilon )p)b}{ \mu +d }}^{S(t)} \biggl(1-\frac{k( \frac{(1-(1-\epsilon )p)b}{ \mu +d})}{k( \sigma )} \biggr)\,d\sigma , \\& V_{2}(t)= \sigma \int _{0}^{h} g( \tau ) \int _{t-\tau }^{t} I(u)\,du\,d\tau , \end{aligned}$$

where $\sigma =\mu +c+\gamma $, and

$$ V_{3}(t)= \int _{0}^{h} g( \tau ) \int _{t-\tau }^{t} T\bigl(I(u)\bigr)\,du\,d\tau . $$

Then

$$ \begin{aligned} \frac{d}{dt}V(t)={}& \biggl(1- \frac{k ( \frac{(1-(1-\epsilon )p)b}{ \mu +d } )}{k(S(t))} \biggr) \biggl(\bigl(1-(1- \epsilon )p\bigr)b- (\mu +d) S(t) \\ &{}- \beta \int _{0}^{h}g(\tau )f\bigl(S(t),I(t-\tau ) \bigr)\,d\tau \biggr) \\ &{} + \beta \int _{0}^{h} g(\tau )f\bigl(S(t),I(t-\tau ) \bigr)\,d\tau -(\mu +c+\gamma )I(t)-T(I) \\ &{}+ \sigma \int _{0}^{h} g( \tau ) \bigl( i(t) - i(t-\tau ) \bigr) \,d\tau + \int _{0}^{h} g( \tau ) \bigl( T\bigl(i(t)\bigr) - T\bigl(i(t-\tau )\bigr)\bigr) \,d\tau \\ = {}& -\mu \biggl(1- \frac{k ( \frac{(1-(1-\epsilon )p)b}{ \mu +d } )}{k(S(t))} \biggr) \biggl(S(t)- \frac{(1-(1-\epsilon )p)b}{ \mu +d } \biggr) \\ &{} + \int _{0}^{h} g(\tau ) \biggl( \beta \frac{k( \frac{(1-(1-\epsilon )p)b}{ \mu +d })}{k(S(t))} \frac{f(S(t),I(t-\tau ))}{I(t -\tau )} -\sigma - \frac{T(I(t-\tau ))}{I(t-\tau )} \biggr)I(t- \tau )\,d\tau . \end{aligned} $$

From hypothesis $(\mathbf{T}_{2})$, it follows that

$$ T^{\prime }(0) \leq \frac{T(I(t-\tau ))}{I(t-\tau )}. $$

Then

$$ \begin{aligned} \frac{d}{dt}V(t) \leq{} & -\mu \biggl(1- \frac{k ( \frac{(1-(1-\epsilon )p)b}{ \mu +d } )}{k(S(t))} \biggr) \biggl(S(t)- \frac{(1-(1-\epsilon )p)b}{ \mu +d } \biggr) \\ &{}+ \int _{0}^{h} g(\tau ) \biggl( \frac{\phi (S(t),I(t - \tau ))}{\sigma +T^{\prime }(0)} \frac{k ( \frac{(1-(1-\epsilon )p)b}{ \mu +d } )}{k(S(t))} -1 \biggr) \bigl(\sigma +T^{\prime }(0) \bigr)I(t - \tau )\,d\tau . \end{aligned} $$

Hypothesis $(\mathbf{H}_{1})$ implies that

$$ -\mu \biggl(1-\frac{k ( \frac{(1-(1-\epsilon )p)b}{ \mu +d } )}{k(S(t))} \biggr) \biggl(S(t)- \frac{(1-(1-\epsilon )p)b}{ \mu +d } \biggr) \leq 0, $$

and hypothesis $(\mathbf{H}_{2})$ gives that

$$ \beta \frac{\phi (S(t),I(t - \tau ))}{\sigma +T^{\prime }(0)} \frac{k ( \frac{(1-(1-\epsilon )p)b}{ \mu +d } )}{k(S(t))} \leq \beta \frac{k(S(t))}{ \sigma +T^{\prime }(0)} \frac{k ( \frac{(1-(1-\epsilon )p)b}{ \mu +d } )}{k(S(t))} = \mathcal{R} _{0}. $$

Hence,

$$ \begin{aligned} \frac{d}{dt}V(t) \leq{} & -\mu \biggl(1- \frac{k ( \frac{(1-(1-\epsilon )p)b}{ \mu +d } )}{k(S(t))} \biggr) \biggl(S(t)- \frac{(1-(1-\epsilon )p)b}{ \mu +d } \biggr) \\ &{}+ ( \mathcal{R}_{0} -1 ) \bigl(\sigma +T^{\prime }(0)\bigr) \int _{0}^{h} g(\tau )I(t-\tau )\,d\tau . \end{aligned} $$

Then the condition $\mathcal{R}_{0} \leq 1$ implies that

$$ \frac{d}{dt}V(t)\leq 0 \quad \mbox{for all } t\geq 0. $$

Moreover, we have

$$ \frac{d}{dt}V(t)=0 \quad \mbox{holds if}\quad (S, I) = (S_{0}, 0). $$

Hence, it follows from system (5) that the set $\{E_{0}\} $ is the largest invariant set in $\{(S, I): \frac{d}{dt}V(t)=0 \}$. By the Lyapunov–LaSalle principle, we conclude that the disease-free equilibrium $E_{0} $ of system (5) is globally asymptotically stable. □

### Global stability of the endemic equilibrium

In this section, we aim to show the global asymptotic stability of the endemic equilibrium $E^{*} $ of system (5) via a Lyapunov stability approach.

#### Theorem 3.4

*If hypotheses* $(\mathbf{H}_{1})$, $(\mathbf{H}_{2})$, $(\mathbf{T}_{1})$, *and* $(\mathbf{T}_{2})$*hold and* $\mathcal{R}_{0} > 1 $, *then the endemic equilibrium of system* (5) *is the only equilibrium and is globally asymptotically stable*.

#### Proof

Let *G* be the function defined from $\mathbb{R}^{+} $ to $\mathbb{R}$ by

$$ G(x)=x-1-\ln (x). $$

It is clear that $G(x)\geq 0 $ if $x > 0$ and $G(x)=0 $ if $x=1 $. Let us consider the following Lyapunov function:

$$ U(t)=U_{1}(t)+U_{2}(t), $$

where

$$ U_{1}(t) = S(t)-S^{*} - \int _{S^{*}} ^{S(t)} \frac{f(S^{*},I^{*})}{f( \sigma ,I^{*})}\,d\sigma + I(t)-I^{*} -I^{*} \ln \biggl(\frac{I(t)}{I^{*}}\biggr) $$

and

$$ U_{2}(t) = \beta f\bigl(S^{*},I^{*}\bigr) \int _{0}^{h} g(\tau ) \int _{t-\tau } ^{t} G\biggl( \frac{ I(U)}{I^{*}} \biggr)\,du\,d\tau . $$

Then

$$ \begin{aligned} \frac{d}{dt}U_{1}(t) ={} & \biggl(1- \frac{f(S^{*},I^{*})}{f(S(t),I^{*})} \biggr) \biggl(\bigl(1-(1-\epsilon )p\bigr)b- (\mu +d) S(t) \\ &{}- \beta \int _{0}^{h} g(\tau )f\bigl(S(t),I(t- \tau ) \bigr)\,d\tau \biggr) \\ &{} + \biggl(1-\frac{I^{*}}{I(t)} \biggr) \biggl(\beta \int _{0}^{h}g(\tau )f\bigl(S(t),I(t-\tau ) \bigr)\,d\tau -(\mu +c+ \gamma )I(t)-T(I) \biggr). \end{aligned} $$

Moreover, we have

$$ \frac{d}{dt}U_{2}(t)= \beta f\bigl(S^{*},I^{*} \bigr) \int _{0}^{h}g(\tau ) \biggl(G\biggl( \frac{I(t)}{I^{*}}\biggr)-G\biggl( \frac{I(t-\tau )}{I^{*}}\biggr) \biggr) \,d\tau $$

and

$$ G\biggl( \frac{I(t)}{I^{*}}\biggr)-G\biggl( \frac{I(t-\tau )}{I^{*}}\biggr)= \frac{I}{I ^{*}}- \frac{I(t- \tau )}{I^{*}} + \ln \biggl( \frac{I(t- \tau )}{I^{*}} \biggr). $$

Since

$$ \textstyle\begin{cases} (1-(1-\epsilon )p)b= (\mu +d) S^{*} + \beta f(S^{*},I^{*}), \\ \beta f(S^{*},I^{*})= ( \mu +c+ \gamma )I^{*}+T(I^{*}), \end{cases} $$

then

$$ \begin{aligned} \frac{d}{dt}U(t)={} & \biggl(1- \frac{f(S^{*},I^{*})}{f(S(t),I^{*})} \biggr) \biggl((\mu +d) S^{*} + \beta f \bigl(S^{*},I^{*}\bigr)- (\mu +d) S(t) \\ &{}- \beta \int _{0}^{h} g(\tau )f\bigl(S(t),I(t- \tau ) \bigr)\,d\tau \biggr) \\ & {}+ \biggl(1-\frac{I^{*}}{I(t)}\biggr) \biggl(\beta \int _{0}^{h}g(\tau )f\bigl(S(t),I(t-\tau ) \bigr)\,d\tau \\ &{}-\beta \frac{ f(S^{*},I ^{*})}{I^{*}}I(t)-T(I)+\frac{I(t)T(I^{*})}{I^{*}} \biggr) \\ &{} + \beta f\bigl(S^{*},I^{*}\bigr) \int _{0}^{h}g(\tau ) \biggl( \frac{I}{I^{*}}- \frac{I(t- \tau )}{I^{*}} + \ln \biggl( \frac{I(t- \tau )}{I^{*}} \biggr) \biggr) \,d\tau \\ ={} & (\mu +d) \biggl(1-\frac{f(S^{*},I^{*})}{f(S(t),I^{*})} \biggr) \bigl(S^{*} - S(t) \bigr) \\ &{} + \beta f\bigl(S^{*},I^{*}\bigr) \int _{0}^{h}g(\tau ) \biggl(1- \frac{f(S^{*},I^{*})}{f(S(t),I^{*})} \biggr) \biggl(1-\frac{f(S(t),I(t- \tau ))}{f(S^{*},I^{*})} \biggr) \,d\tau \\ &{} + \beta f\bigl(S^{*},I^{*}\bigr) \int _{0}^{h}g(\tau ) \biggl(1- \frac{I^{*}}{I(t)}\biggr) \biggl(\frac{f(S(t),I(t- \tau ))}{f(S^{*},I^{*})} - \frac{I(t)}{I^{*}} \biggr)\,d\tau \\ &{} + \beta f\bigl(S^{*},I^{*}\bigr) \int _{0}^{h}g(\tau ) \biggl( \frac{I}{I^{*}}- \frac{I(t- \tau )}{I^{*}} + \ln \biggl( \frac{I(t- \tau )}{I^{*}} \biggr) \biggr) \,d\tau \\ &{}+ \biggl(1-\frac{I^{*}}{I(t)} \biggr) \biggl( \frac{I(t)T(I^{*})}{I^{*}}-T(I) \biggr). \end{aligned} $$

It follows that

$$\begin{aligned} \frac{d}{dt}U(t) ={}& (\mu +d) \biggl(1- \frac{f(S^{*},I^{*})}{f(S(t),I ^{*})} \biggr) \bigl(S^{*} - S(t) \bigr) \\ &{} + \beta f\bigl(S^{*},I^{*}\bigr) \int _{0}^{h}g(\tau ) \biggl(2- \frac{f(S^{*},I^{*})}{f(S(t),I^{*})} + \frac{f(S(t),I(t- \tau ))}{f(S(t),I^{*})} \\ &{}- \frac{I^{*}}{I(t)} \frac{f(S(t),I(t- \tau )}{f(S^{*},I^{*})} \biggr) \,d\tau \\ &{} + \beta f\bigl(S^{*},I^{*}\bigr) \int _{0}^{h}g(\tau ) \biggl( - \frac{I(t- \tau )}{I^{*}} + \ln \biggl( \frac{I(t- \tau )}{I^{*}}\biggr) \biggr)\,d\tau \\ &{}+ \biggl(1-\frac{I^{*}}{I(t)} \biggr) \biggl(\frac{I(t)T(I^{*})}{I^{*}}-T(I) \biggr). \end{aligned}$$

Using

$$\begin{aligned} \ln \biggl( \frac{I(t- \tau )}{I^{*}}\biggr) =& \ln \frac{f(S^{*},I^{*})}{f(S(t),I ^{*})} + \ln \biggl(\frac{I^{*}}{I(t)} \frac{f(S(t),I(t- \tau ))}{f(S ^{*}, I^{*})} \biggr) \\ &{}+\ln \biggl( \frac{I(t- \tau )}{I^{*}} \frac{f(S(t),I ^{*})}{f(S(t),I(t- \tau ))} \biggr), \end{aligned}$$

it follows that

$$ \begin{aligned} \frac{d}{dt}U(t)={} & (\mu +d) \biggl(1- \frac{f(S^{*},I^{*})}{f(S(t),I ^{*})} \biggr) \bigl(S^{*} - S(t) \bigr) \\ &{} + \beta f\bigl(S^{*},I^{*}\bigr) \int _{0}^{h}g(\tau ) \biggl(1- \frac{f(S^{*},I^{*})}{f(S(t),I^{*})} + \ln \frac{f(S^{*},I^{*})}{f(S(t),I^{*})} \biggr) \,d\tau \\ &{} + \beta f\bigl(S^{*},I^{*}\bigr) \int _{0}^{h}g(\tau ) \biggl(1- \frac{I^{*}}{I(t)} \frac{f(S(t),I(t- \tau ))}{f(S^{*},I^{*})} \\ &{}+ \ln \biggl( \frac{I^{*}}{I(t)} \frac{f(S(t),I(t- \tau ))}{f(S^{*},I^{*})} \biggr) \biggr)\,d\tau \\ &{} + \beta f\bigl(S^{*},I^{*}\bigr) \int _{0}^{h}g(\tau ) \biggl(1- \frac{i(t- \tau )}{I^{*}} \frac{f(S(t),I ^{*})}{f(S(t),I(t- \tau ))} \\ &{}+ \ln \biggl( \frac{I(t- \tau )}{I^{*}} \frac{f(S(t),I ^{*})}{f(S(t),I(t- \tau ))} \biggr) \biggr)\,d\tau \\ &{} + \beta f\bigl(S^{*},I^{*}\bigr) \int _{0}^{h}g(\tau ) \biggl( \frac{I(t- \tau )}{I^{*}} \frac{f(S(t),I ^{*})}{f(S(t),I(t- \tau ))} - 1 \\ &{}- \frac{I(t- \tau )}{I^{*}} + \frac{f(S(t),I(t- \tau ))}{f(S(t),I^{*})} \biggr) \,d\tau \\ &{} + \bigl(I(t)-I^{*} \bigr) \biggl(\frac{T(I^{*})}{I^{*}}- \frac{T(I)}{I(t)} \biggr). \end{aligned} $$

By hypotheses $(\mathbf{H}_{1})$ and $(\mathbf{H}_{2})$ we have

$$ \begin{aligned} &\frac{I(t- \tau )}{I^{*}} \frac{f(S(t),I^{*})}{f(S(t),I(t- \tau ))} - 1- \frac{I(t- \tau )}{I^{*}} + \frac{f(S(t),I(t- \tau ))}{f(S(t),I ^{*})} \\ &\quad = \biggl( \frac{I(t- \tau )}{I^{*}} - \frac{f(S(t),I(t- \tau ))}{f(S(t),I ^{*})} \biggr) \biggl( \frac{f(S(t),I^{*})}{f(S(t),I(t- \tau ))}-1 \biggr) \\ &\quad = \frac{I(t- \tau )}{I^{*} \phi (S(t),I^{*}) f(S(t),I(t-\tau ))} \bigl(\phi \bigl(S(t),I^{*}\bigr)-\phi \bigl(S(t),I(t-\tau )\bigr) \bigr) \\ &\qquad {}\times\bigl(f\bigl(S(t),I ^{*}\bigr)-f \bigl(S(t),I(t-\tau )\bigr) \bigr), \end{aligned} $$

and then

$$ \frac{I(t- \tau )}{I^{*}} \frac{f(S(t),I^{*})}{f(S(t),I(t- \tau ))} - 1- \frac{I(t- \tau )}{I^{*}} + \frac{f(S(t),I(t- \tau ))}{f(S(t),I ^{*})}\leq 0. $$

Moreover, hypothesis $(\mathbf{H}_{1})$ implies that

$$ (\mu +d) \biggl(1-\frac{f(S^{*},I^{*})}{f(S(t),I^{*})} \biggr) \bigl(S^{*} - S(t) \bigr) \leq 0, $$

and hypothesis $(\mathbf{T}_{2})$ gives

$$ \bigl(I(t)-I^{*} \bigr) \biggl(\frac{T(I^{*})}{I^{*}}- \frac{T(I)}{I(t)} \biggr)\leq 0. $$

Hence, $\frac{d}{dt}U(t) \leq 0$. We conclude that the endemic equilibrium of system (5) is globally asymptotically stable. □

## Numerical results

In this section, we present the numerical simulation of the model by considering the following delayed SIR epidemic model with vaccination, treatment, and distributed time delay:

12 $$ \textstyle\begin{cases} \frac{dS(t)}{dt}=(1-(1-\epsilon )p)b- (\mu +d) S(t)- \beta \int _{0}^{h} \frac{ e^{- \tau }}{1- e ^{-h}}S(t)I(t-\tau ) \,d\tau , \\ \frac{dI(t)}{dt}= \beta \int _{0}^{h} \frac{ e^{- \tau }}{1- e^{-h}}S(t)I(t- \tau ) \,d\tau -( \mu +c+ \gamma )I(t)-\frac{a I(t)}{1+ \xi I(t)}, \\ \frac{dR(t)}{dt}=(1-\epsilon )pb+\gamma I(t)+ \frac{a I(t)}{1+\xi I(t)} +d S(t)- \mu R(t). \end{cases} $$

The function *g* is chosen, as in [37], in the following form:

$$ g( \tau ) = \frac{ e^{- \tau }}{1- e ^{-h}}. $$

On the other hand, the treatment function *T*, similarly to [23], is defined by

$$ T(I)= \frac{a I}{1+\xi I}. $$

The reproduction number $\mathcal{R}_{0}$ is given by

13 $$ {\mathcal{R}}_{0} = \frac{ \beta (1-(1-\epsilon )p)b}{ (\mu +d)( \mu + c + \gamma +a)}. $$

For our system (12) without vaccination and treatment, the reproduction number is given by

$$ \overline{\mathcal{R}_{0}} = \frac{ \beta b}{ \mu (\mu +c +\gamma )}. $$

Hence $\mathcal{R}_{0} $ can be rewritten as

$$ \mathcal{R}_{0} = \frac{\mu (\mu +c +\gamma )(1-(1-\epsilon )p)}{( \mu +d)(\mu +c +\gamma +a)}\overline{ \mathcal{R}_{0}}. $$

If $\overline{\mathcal{R}_{0}} \leq 1 $, then the disease will die out (the disease-free equilibrium $E_{0}$ is globally asymptotically stable) without any control measures.

However, if $\overline{\mathcal{R}_{0}} > 1$, then

$$ \mathcal{R}_{0}\leq 1 \quad \mbox{is equivalent to}\quad S_{0} \leq \bar{S}= \frac{\mu +c +\gamma +a}{\beta }, $$

where $S_{0}$ is given in (6). Similarly,

$$ \mathcal{R}_{0}\geq 1\quad \mbox{is equivalent to}\quad S_{0} \geq \bar{S} . $$

This shows that, during the epidemic $\overline{\mathcal{R}_{0}} > 1 $, if the number of susceptible population is below the threshold *S̄*, then the disease can be controlled by vaccination and treatment. However, if the number of susceptible population is above the threshold *S̄*, then the disease will persist in the population.

To make sense of our simulation, we will focus on the case of $\overline{\mathcal{R}_{0}} > 1$, and we choose the parameters *p* and *a* to guarantee the clearance of the disease from the population by these two public health control measures.

We consider the following initial conditions:

$$\begin{aligned}& \Phi _{1}(\theta )=\sin (0.5 \theta ) + 100,\qquad \Phi _{2}( \theta ) = \sin (10 \theta ) + 20, \\& \Phi _{3}(\theta ) = 0\quad \mbox{for } -h\leq \theta \leq 0, \\& \Phi _{1}(\theta )=\cos (5 \theta ) + 200,\qquad \Phi _{2}( \theta ) = 10 \cos ( \theta ) + 30, \\& \Phi _{3}(\theta ) = 0\quad \mbox{for } -h\leq \theta \leq 0, \\& \Phi _{1}(\theta )=\cos (5 \theta ) + 260,\qquad \Phi _{2}( \theta ) = 30+20 \sin (10 \theta ) , \\& \Phi _{3}(\theta ) = 80\quad \mbox{for } -h\leq \theta \leq 0, \\& \Phi _{1}(\theta )=\cos (5 \theta ) + 280,\qquad \Phi _{2}( \theta ) = 30+40 \sin (10 \theta ) , \\& \Phi _{3}(\theta ) = 30\quad \mbox{for } -h\leq \theta \leq 0, \\& \Phi _{1}(\theta )=\cos (5 \theta ) + 300,\qquad \Phi _{2}( \theta ) = 30+70 \sin (10 \theta ) , \\& \Phi _{3}(\theta ) = 50\quad \mbox{for } -h\leq \theta \leq 0. \end{aligned}$$

All the numerical simulations are performed using the explicit Runge–Kutta-like method (dde45) [38].

**Figure 3:**
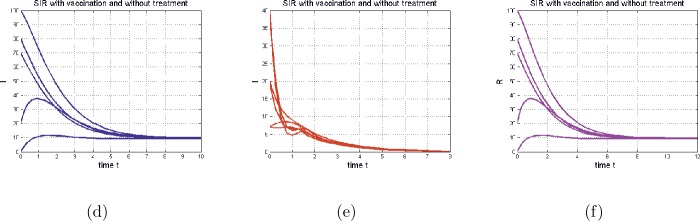
The time series of model (3) in the special case (12), with Figures (**d**), (**e**), and (**f**) representing (respectively) $S(t)$, $I(t)$, and $R(t)$. The parameters of the model are $b = 10$, $\mu = 0.65 $, $\beta = 0.2 $, $c = 0.77 $, $\gamma = 0.75 $, $h=1.5$, $d=0.4$, $p=0.4$, $\epsilon =0.2$, $\xi = 10$, and $a=0$. In this case $\mathcal{R}_{0}=0.5969< 1$

**Figure 4:**
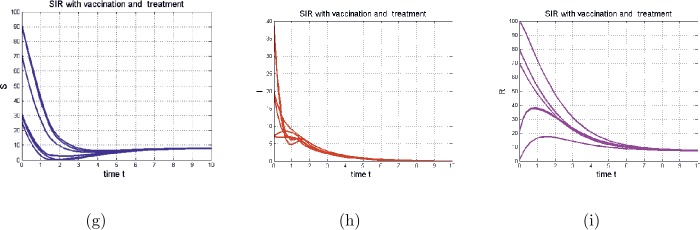
The time series of model (3) in the special case (12), with Figures (**g**), (**h**), and (**i**) representing (respectively) $S(t)$, $I(t)$, and $R(t)$. With the same parameters as in Fig. 3 except $p=0.3$, $\epsilon =0.2$ and $d=0.3$ and $a=0.5$. In this case, $\mathcal{R}_{0}=0.5993< 1$

First, we start with the case of no vaccination and no treatment ($p=0$ and $a=0$). In this situation our model is similar to that of Enatsu et al. [10], in which the authors claim that when the basic reproduction number, denoted by $\overline{\mathcal{R}_{0}}$, is greater than one ($\overline{ \mathcal{R}_{0}} > 1$), the disease persists. However, our numerical simulation (Fig. 3 and Fig. 4) shows that the disease will die out even if $\overline{\mathcal{R}_{0}}>1$.

Next, we consider the case with vaccination and no treatment, with $\overline{\mathcal{R}_{0}} > 1$ and $\mathcal{R}_{0} < 1$. As shown in Fig. 3, the disease dies out, which corresponds to our theoretical result.

Finally, we give the simulation of the case with vaccination and treatment, with $\overline{\mathcal{R}_{0}} > 1$ and $\mathcal{R}_{0} < 1$. As shown in Fig. 4, the treatment with vaccination helps the eradication of the infection from the population.

For more illustration, it is very interesting to discuss the behavior of the basic reproduction number $R_{0}$ with respect to vaccination and treatment parameters. Namely, the parameters *p*, *d*, and *a*. From the expression of $R_{0}$, formula (13), it is clear that $R_{0}$ is a decreasing function with respect to *p*, *d*, and *a* respectively on $[0,1]$, $[0,1]$, and $[0, +\infty [$. Moreover, $R_{0}$ is an equation of a straight line with respect to *p* and

$$ \lim_{a \longrightarrow + \infty }R_{0}(a )= 0. $$

**Figure 5:**
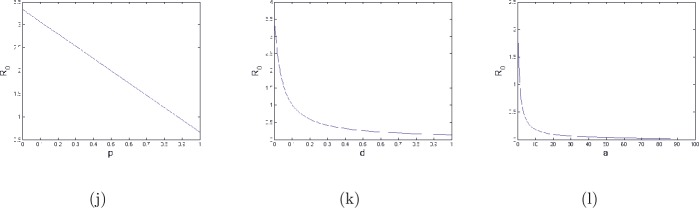
The behavior of $R_{0}$ in the special case (12) with the parameters: $b = 10$, $\mu = 0.04 $, $\beta = 0.15 $, $c = 0.5 $, $\gamma = 0.003 $, $d=0.5$, $p=0.8$, $\epsilon =0.2$, and $a=0.3$ for (**j**) and the same parameters except $b=20$ for (**k**) and (**l**)

In Fig. 5 we show the effect of vaccination and treatment parameters on the dynamic of $R_{0}$. We notice that the critical values $\bar{p}=0.875$, $\bar{d}=1.02$, and $\bar{a}=1.157$ represent separated values between the endemic state and the disease-free state for (j), (k), and (l) respectively (it means the cases $R_{0}<1$ and $R_{0}>1$).

## Conclusion

In this work, we analyzed a delayed SIR model with generalized incidence function and distributed delay as the contact between infected individuals and healthy ones does not result in an immediate infection. The delay, presented in this work, reflects the time that it takes to have an infection after the contact. The model also included the two main types of disease control measures: vaccine and treatment. The question that arises in using these two measures is how each vaccination should depend on the treatment. In fact, as the treatment is the first control measure to be taken either as a prophylactic or antiviral, the vaccination implementation should take into consideration the effect of the treatment on the disease infectiousness. Moreover, the function treatment was chosen to reflect the reality of drug stock supply during the time of the infections. Our analysis showed that when $\mathcal{R}_{0} \leq 1$, the disease-free equilibrium is globally asymptotically stable, and when $\mathcal{R}_{0} > 1$, then there is a unique disease-endemic equilibrium, which is globally asymptotically stable. To put this result in context, we chose the treatment function $T(I)= \frac{a I(t)}{1+ \xi I(t)}$ (see [23]).

In our analysis we showed that when the disease is endemic, in the absence of the vaccination and treatment, then there are two possible scenarios: (a) if the number of susceptible population is below the threshold *S̄*, then the disease can be controlled by vaccination and treatment; (b) if the susceptible population is above the threshold *S̄*, then the disease will persist in the population. This finding reflects the limited capability of the control measure to eradicate the disease if the population is too large.
